# Childhood trauma in patients with Schizophrenia and schizoaffective disorder

**DOI:** 10.1192/j.eurpsy.2026.12185

**Published:** 2026-07-31

**Authors:** E. Maaloul, R. Chakchouk, S. Ellouze, F. Sahnoun, N. Halouani, M. Turki, J. Aloulou

**Affiliations:** Psychiatry « B » Department, CHU Hedi Chaker Sfax, Sfax, Tunisia

## Abstract

**Introduction:**

Schizophrenia and schizoaffective disorder are complex psychiatric conditions involving multiple biological, psychological, and environmental determinants. Early traumatic experiences are increasingly recognized as significant vulnerability factors that may shape symptom profiles, influence treatment outcomes, and affect overall functional prognosis.

**Objectives:**

This study aims to determine the prevalence of childhood trauma in this clinical population and to identify the main associated factors that may contribute to the variability of clinical presentation and disease outcomes.

**Methods:**

A cross-sectional study was conducted among thirty-one outpatients meeting diagnostic criteria for schizophrenia or schizoaffective disorder. Sociodemographic characteristics, clinical features, and treatment-related variables were obtained through structured interviews and review of medical records. Exposure to early adverse experiences was evaluated using the Childhood Trauma Questionnaire – Short Form (CTQ-SF).

**Results:**

The study sample consisted of 31 participants (48.4% men) with a mean age of 38.1 ± 9.3 years. The mean age at illness onset was 26.2 ± 7.6 years, with an average of 3.8 ± 3.8 hospitalizations. Most participants were unemployed (71%), single (71%), and had a chronic illness course exceeding 10 years (45.2%). Schizophrenia was diagnosed in 58.1% of casesand schizoaffective disorder in 41.9% of cases.Regarding treatment, 67.7% were receiving classicantipsychotics, 41.9% atypical agents, and 58% were on long-acting injectable formulations. Clozapine was prescribed in 6.5% of cases, corresponding to treatment-resistant profiles. The mean CTQ total score was 56, and 77.4% of patients reported at least one form of childhood maltreatment. The most frequent trauma subtypes were emotional abuse (13.5 ± 5.2) and emotional neglect (13.4 ± 4.3), reflecting predominant emotional adversity. The total CTQ score was significantly associated with the number of hospitalizations and the duration of illness.

**Conclusions:**

The high prevalence of childhood trauma within this population, and its significant associations with the number of hospitalizations and illness duration, suggests that early adverse experiences may contribute to illness chronicity and poorer clinical trajectories. These findings underscore the importance of trauma-informed and multidisciplinary interventions combining pharmacological optimization, adherence reinforcement, and psychosocial rehabilitation to improve long-term outcomes.

**Disclosure of Interest:**

None Declared

